# Supplementary material for: Whole Goat Milk-Based Formula versus Whey-Based Cow Milk Formula: What Formula Do Infants Enjoy More?—A Feasibility, Double-Blind, Randomized Controlled Trial
Source: Nutrients. 2023 Sep 19;15(18):4057. doi: 10.3390/nu15184057 (PMC10537215; doi:10.3390/nu15184057)
Supplement: Supplementary file 1 [file nutrients-15-04057-s001.zip › nutrients-2595606-supplementary.pdf]

Table S1

		GMF (Capricare)	CMF
Nutrient	Unit	per 100 mL of prepared feed	per 100 mL of prepared
**Energy**	kJ	280	280
	kcal	67	67
**Protein**	g	1.3	1.3
Whey: casein ratio		20:80	60:40
**Carbohydrate**	g	7.6	7.6
Of which lactose	g	7.6	7.6
**Fat**	g	3.5	3.5
Milk fat	%	48	22
**Docosahexaenoic acid (DHA)**	mg	9.4	9.4
**Arachidonic acid (AA)**	mg	10	10
**Vitamins**			
Vitamin A	µg-RE	71	71
Vitamin D_3	µg	1	1
Vitamin E	mg α-TE	1.6	1.6
Vitamin K_1	µg	6.9	6.9
Vitamin C	mg	14	14
Vitamin B_1	mg	0.06	0.06
Vitamin B_2	mg	0.13	0.13
Niacin	mg	0.7	0.7
Vitamin B_6	µg	0.04	0.04
Folic acid	µg	8.8	8.8
Pantothenic acid	mg	0.36	0.36
Vitamin B_{12}	µg	0.18	0.18
Biotin	µg	2.4	2.4
**Minerals**			
Sodium	mg	18	18
Potassium	mg	75	75
Chloride	mg	70	70
Calcium	mg	66	66
Phosphorus	mg	43	43
Magnesium	mg	6.1	6.1
Iron	mg	0.72	0.72
Zinc	mg	0.5	0.5
Copper	mg	0.05	0.05
Manganese	mg	0.01	0.01
Fluoride	µg	6.5	6.5
Selenium	µg	1.3	1.3
Iodine	µg	8.3	8.3
**Other nutrients**			
Choline	mg	13	13
Taurine	mg	4.7	4.7
Carnitine	mg	0.92	0.92
Inositol	mg	4	4

Table S2

Your child . . .	Definitely false	Mostly false	Both true and false	Mostly true	Definitely true	I don’t know
1. eats well	☐	☐	☐	☐	☐	☐
2. looks well	☐	☐	☐	☐	☐	☐
3. is mentally alert	☐	☐	☐	☐	☐	☐
4. is restless	☐	☐	☐	☐	☐	☐
5. likes to be looked after	☐	☐	☐	☐	☐	☐
6. is happy, quick to smile or laugh	☐	☐	☐	☐	☐	☐
7. always needs to be looked after	☐	☐	☐	☐	☐	☐
8. has a good family environment	☐	☐	☐	☐	☐	☐
9. is full of life and energy	☐	☐	☐	☐	☐	☐
10. cries when left alone	☐	☐	☐	☐	☐	☐
11. adapts easily to change	☐	☐	☐	☐	☐	☐
12. babbles freely	☐	☐	☐	☐	☐	☐
13. often wants to be picked up	☐	☐	☐	☐	☐	☐
14. is cheerful	☐	☐	☐	☐	☐	☐
15. likes seeing people	☐	☐	☐	☐	☐	☐
16. has parents who get along with each [illegible]	☐	☐	☐	☐	☐	☐
17. sleeps well	☐	☐	☐	☐	☐	☐
18. cries often	☐	☐	☐	☐	☐	☐
19. is cuddly and affectionate	☐	☐	☐	☐	☐	☐
20. cries for reasons easily understood	☐	☐	☐	☐	☐	☐
